# Evaluation of hearing preservation in adults with a slim perimodiolar electrode

**DOI:** 10.1007/s00405-021-06755-z

**Published:** 2021-04-08

**Authors:** Sonja Ludwig, Niklas Riemann, Stefan Hans, Florian Christov, Johannes Maximilian Ludwig, Judith Saxe, Diana Arweiler-Harbeck

**Affiliations:** 1 Department of Otorhinolaryngology, Head and Neck Surgery, Medical Faculty Mannheim, University Hospital Mannheim, University of Heidelberg, Theodor-Kutzer-Ufer 1-3, Mannheim, Germany; 2 Department of Otorhinolaryngology, Head and Neck Surgery, University Hospital Essen, University of Duisburg-Essen, Essen, Germany; 3 ENT Practice Cologne-Bonn, Wesseling, Germany; 4 Department of Diagnostic and Interventional Radiology and Neuroradiology, University Hospital Essen, University of Duisburg-Essen, Essen, Germany

**Keywords:** Cochlear implant, Slim perimodiolar electrode, Hearing preservation, Residual hearing

## Abstract

**Purpose:**

Numerous endeavors have been undertaken to preserve hearing in cochlear implant (CI) patients. Particularly, optimization of electrode array design aims at preservation of residual hearing (RH). This study examines whether a slim perimodiolar (PM) electrode array could bear the capability to preserve hearing.

**Methods:**

A total of 47 patients underwent cochlear implantation receiving the PM electrode. (i) Patients with pure tone audiogram (PTA) thresholds better than 85 dB and/or hearing loss for Freiburg speech test numbers less than 60 dB and more than 50% maximum monosyllabic understanding were assigned to the RH group (*n* = 17), while all others belonged to the noRH group (*n* = 30). (ii) Another group implanted with a slim straight, lateral wall (LW) electrode was recruited for comparison.

**Results:**

We compared 17 RH–30 noRH patients all receiving the PM electrode. RH in PM recipients decreased faster than in LW recipients. No significant differences were observed between both (RH v/s noRH) groups in NRT thresholds, Freiburg speech test and A§E^®^ phonemes. Analogous satisfaction levels were indicated through the questionnaires in terms of sound quality, hearing in silence, noise and directional hearing in both groups.

**Conclusions:**

The results suggest that hearing preservation is influenced not only by electrode shape but various factors. This study opens an avenue for further investigations to elucidate and enumerate the causes for progressive hearing loss.

**Supplementary Information:**

The online version contains supplementary material available at 10.1007/s00405-021-06755-z.

## Introduction

Cochlear implantation is an effective treatment option for profound sensorineural hearing loss. Compared to the first cochlear implants in the 1980′s, surgical techniques and implant technologies have evolved over the past decades [1]. The possibility of hearing preservation (HP) after cochlear implantation is a novel goal: The indication criteria for cochlear implantations have expanded including more patients with residual hearing, which turns HP into a favorable therapeutic target [2]. In general, HP in cochlear implant patients aims at an electrically and acoustically mediated hearing perception, which allows for an optimized speech understanding in complex hearing situations such as noise, sound localization and music perception. Multiple insertion trauma reducing factors are considered to protect residual hearing after implantation: surgical techniques, electrode array design and intracochlear administration of pharmacological agents (i.e., corticosteroids) [3–7]. Novel technical developments target fibrosis and scar prevention using corticosteroid hydrogels, corticosteroid-eluting or nanoparticle-covered cochlear implant arrays [8]. Also, partial insertion is discussed as a legitimate method for HP in high-frequency hearing loss [9]. New technologies promote robot-assisted surgeries to scale down the insertion speed and reduce the trauma [10].

However, to date there is no consensus about which factors are critical for hearing preservation. Some subjects eventually lose their residual hearing in long term [11–13]. Also, the types of electrode arrays are controversially discussed: perimodiolar (precurved, PM) or lateral wall (straight, LW). New electrode arrays have advanced to be thinner, more delicate and tend to a closer electrode-neuron interface addressing efficient signal transmission [14].

A slim, precurved modiolar electrode has been reported having combined advantages over previous cochlear implants by its precurved design, flexible-thin character and is supposedly beneficial for HP in particular.

Thus, the main objective of this study is to evaluate the possibility of hearing preservation within PM recipients. To address this objective we compared (i) the PM recipients with residual hearing (RH) prior to implantation directly to patients with noRH, and additionally (ii) the PM recipients to LW recipients to examine the role of electrode shape in hearing preservation. As the PM electrode is thinner than the LW electrode and precurved, our study hypothesis is that PM recipients might show better hearing preservation in adults.

## Materials and methods

### Patients

All 47 subjects received the Nucleus Slim Modiolar Electrode (CI532, Cochlear^®^/PM electrode) at our department of Otorhinolaryngology between 9/2016 and 11/2018 after informed consent. The study was approved by the local ethics committee and was conducted in accordance to the Declaration of Helsinki. The inclusion criteria were defined as following: firstly, minimum age of 18 years at implantation. Secondly, native German language skills were required (both verbal and written) to answer the questionnaires as well as for participating in Freiburg speech test. Depending on the level of residual hearing prior to implantation subjects were split into 2 groups: 17 patients with residual hearing (RH) and 30 patients without residual hearing (noRH). RH was defined according to the following criteria: (i) hearing threshold better than ( <) 85 dB for pure tone audiogram (PTA) frequencies 250–500–1000–2000 Hz, and/or (ii) hearing loss for Freiburg speech test numbers of less than 60 dB and more than 50% of maximum Freiburg monosyllables understanding (40 items each). All study participants gave written informed consent prior to study inclusion. For comparison, data from 26 patients with a Slim Straight electrode (CI522, Cochlear^®^/LW) were compared to the PM recipients. Patients who were unable to attend their regular check-up appointments either because of missing, declining or postponing their appointments or relocating post-implantation were summarized as “loss of follow up”.

### PM electrode and surgical technique

The Nucleus^®^ CI532 cochlear implant with slim modiolar electrode and the CochlearTM Nucleus^®^ CI522 cochlear implant with Slim Straight electrode were inserted according to the manufacturer’s instructions by two experienced surgeons routinely performing cochlear implantations: (i) The CI532 implant is precurved and secured in a thin sheath to hold it in straight position aiming at perimodiolar position. Due to the greater diameter of the insertion tool in all cases, it required an extended round window or a combined (round window/cochleostomy) approach. (ii) The CI522 implant consists of a slim straight electrode aiming at a lateral wall position. All CI522 electrodes could be inserted by a round window approach, as there is no additional insertion tool.

Both electrodes were inserted into the cochlea by slowly advancing the electrode until the white markers were aligned. For the CI532 insertion, the sheath is pulled back by its grip and the three markers of the electrode were kept in position in the cochlear opening. Intraoperatively all subjects received 250 mg prednisolone intravenously shortly before insertion.

### Imaging

To ensure the correct position of the electrode ConeBeam, Computed Tomography (CT) (intraoperatively) or regular CT scan (postoperatively directly after surgery) was conducted. Electrode position was evaluated to determine the Wrapping factor (WF) according to Holden et al. [15]. WF is calculated as a quotient of electrode trajectory length (*L*_EL_) and lateral wall length (*L*_LW_) (*WF* = *L*_EL_*/L*_LW_). The smaller the WF value becomes, the closer the electrode is aligned to the modiolus of the cochlea (*L*_EL_ < *L*_LW_). WF values of the PM electrode were related to the LW electrode.

### Audiometry

#### Pure-tone audiometry (PTA)

Residual hearing levels were compared by unaided pure-tone audiometry (PTA). Hearing thresholds were detected for different frequencies by bone and air conduction (250–500–1000–2000 Hz) before surgery, on the first day, 3–6–12 months post-surgery in a regular follow-up setting. Particularly, bone conduction thresholds were compared to analyze inner ear functions. Bone conduction values on the first day post-surgery were inconsistent due to different setups and bandages, hence these values were neglected for further analysis. If no detectable threshold for the tested frequency was observed, hearing threshold was visualized as > 110 dB in the PTA graphs.

#### Neural response telemetry (NRT)

Objective audiometry was measured as electrically evoked compound action potentials (ECAPs) in neural response telemetry (NRT) as reported previously [16]. Briefly after electrode insertion and in the follow-up period, ECAP thresholds were detected using the AutoNRT program provided by Cochlear [17]. AutoNRTs were measured to compare the ECAP threshold as an objective measure of the quantity of neural stimulation by the electrode. These threshold tNRTs are calculated in current level (CL) units based on a linear regression model and allow a tonotopic resolution of the stimulation of the cochlea. The 22 electrode segments (ES) are combined in total and 3 subunits according to the localization in the cochlea: apical (22–16 ES), medial (15–8 ES), basal (7–1 ES). For this analysis, LW and PM recipients were matched for their RH prior to surgery. Mean thresholds of AutoNRTs are shown as boxplots in current levels.

#### Freiburg speech recognition test

Freiburg speech recognition was tested for 50% recognition level of numbers and maximum of monosyllabic words at 65 dB as first described by Hahlbrock et al. [18]. The implanted subjects were tested wearing the cochlear implant while masking the contralateral ear prior to surgery and in the course of the follow-up (3–6–12 months).

#### Phoneme detection and discrimination in A§E^®^

A§E^®^, developed by The Eargroup (Antwerp, Belgium), was assessed to compare speech sounds in best aided conditions before cochlear implantation and during follow-up. Best aided condition was attained by wearing hearing aids and/or CIs during the hearing test. The hearing aids were validated and optimized prior to cochlear implantation by an experienced hearing care professional. In general, A§E^®^ is a useful tool for frequency-dependent fitting in cochlear implant patients that is more independent from age, cognitive level and language skills than other speech tests (i.e., Freiburg speech test) [19]. Speech sounds were measured as phoneme discrimination and detection levels (ranging from 0 to 100%). In the follow-up period, the test was repeated until 100% was reached.

### Questionnaires

Subjects were asked to complete the questionnaires “HISQUI19” and Oldenburg Inventory prior to the implantation, 3, 6 and 12 months after implantation.

To evaluate the subjective sound quality in patients, the questionnaire ‘Hearing Implant Sound Quality Index’ (HISQUI19, MEDEL^®^) was applied. The subjects filled out a form containing 19 questions. Each of the question had a score ranging from 1 (never) − 7 (always). Upon aggregation, the scores were categorized into: < 31: very bad sound quality, 31–60: bad sound quality, 61–90: medium sound quality, 91–110: good sound quality, 111–133: very good sound quality.

Oldenburg Inventory (“Oldenburger Inventar-R”) is a questionnaire requiring patients’ evaluation on subjective abilities to hear in daily life with hearing aids/implants. Patients were asked to gauge their understanding (never-rarely-sometimes-often-always) for the situation at home, work and while interacting with others. Each question (32 in total) represents a certain hearing condition in silence, noise and direction. For the statistical analysis, scores ranging between 1 and 5 were distributed for each condition and related to the maximum score (= 100%).

### Statistical analysis

Data analysis was performed using GraphPad Prism 7 for Mann–Whitney test. *p* values below 0.05 were considered to be significant.

## Results

Patients’ clinical characteristics are summarized in Table 1. Overall, 47 patients received the PM electrode. Mean age of the subjects was 51, ranging from 19 to 86 years. The PM recipients consisted of 23 males and 24 females. The etiology of hearing loss was in most cases presbyacusis or idiopathic. Hereditary, infectious or Meniere’s disease were amongst the less frequent reasons for hearing loss. Two patients were implanted bilaterally. 40 patients had a bilateral, symmetric hearing loss, in seven cases the hearing loss was asymmetric. 17 patients fulfilled the criteria for RH and 30 patients belonged to the noRH group. 30 right ears and 19 left ears were implanted.

**Table 1:** Clinical characteristics of patients implanted with a PM and LW electrode

Patient characteristics				
	CI532		CI522	
	*n*	%	*n*	%
No. Patients	47	100	26	100
Age (Mean; years)	19–86 years		21–83 years	
≤51	22	50	15	58
> 51	25	50	11	42
Gender				
Male	23	50	13	50
Female	24	50	13	50
Residual hearing (RH)				
RH	17	36	19	73
No RH	30	64	7	27
Hearing loss				
Symmetric	40	85	24	92
Asymmetric	7	15	2	8
Etiology				
Presbyacusis	16	34	11	42
Hereditary	7	15	3	12
Infection	5	11	1	4
Menière ‘s disease	1	2	1	4
Idiopathic	18	38	10	38
No. Implants	49	100	26	100
Localization				
Left	19	38	18	69
Right	30	61	8	31

The LW recipients were matched for age and gender: 19 patients belonged to the RH group and 7 patients to the noRH group according to the above defined criteria. Most patients (92%) suffered from symmetric hearing loss and presbyacusis/idiopathic hearing loss. 18 CIs were implanted in the left ear and eight CIs in the right ear.

**Fig. 1:**
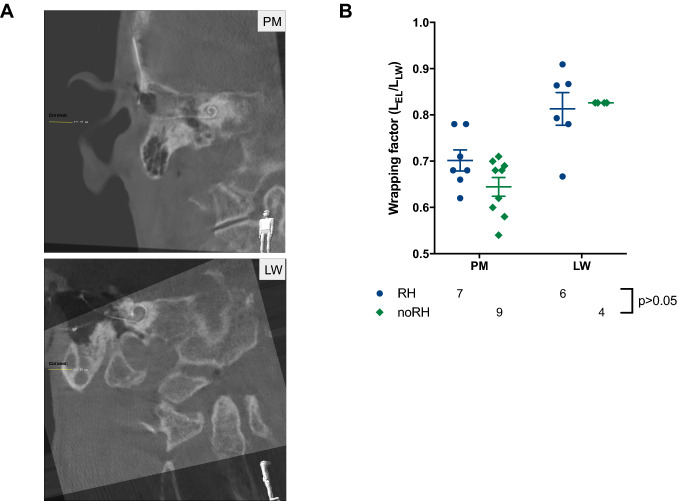
Cone beam Computed Tomography (CT) or regular CT scans were conducted for all cochlear implant patients to ensure a correct position of the array in the cochlea. **a** CT scan of the PM CI532 electrode (left) and LW electrode (right) in the cochlea. **b** Wrapping factors of the PM electrode in relation to the LW electrode for both RH vs. noRH groups (prior to implantation)

CT scan images showed a proper localization of the PM electrode in the scala tympani of the cochlea in almost all cases (Fig. 1a). In two cases, the electrode was bent in a tip fold over. One patient had to be reoperated immediately because of a complete tip fold over. The electrode was replaced by a LW electrode. This patient was excluded from the study. Analysis of the WF in CT scans summed up to average of 0.67 (noRH 0.64 vs. RH 0.7) and confirmed the perimodiolar localization of the PM electrode (Fig. 1b). Mean WF for the LW electrodes was around 0.82 (noRH = RH).

**Fig. 2:**
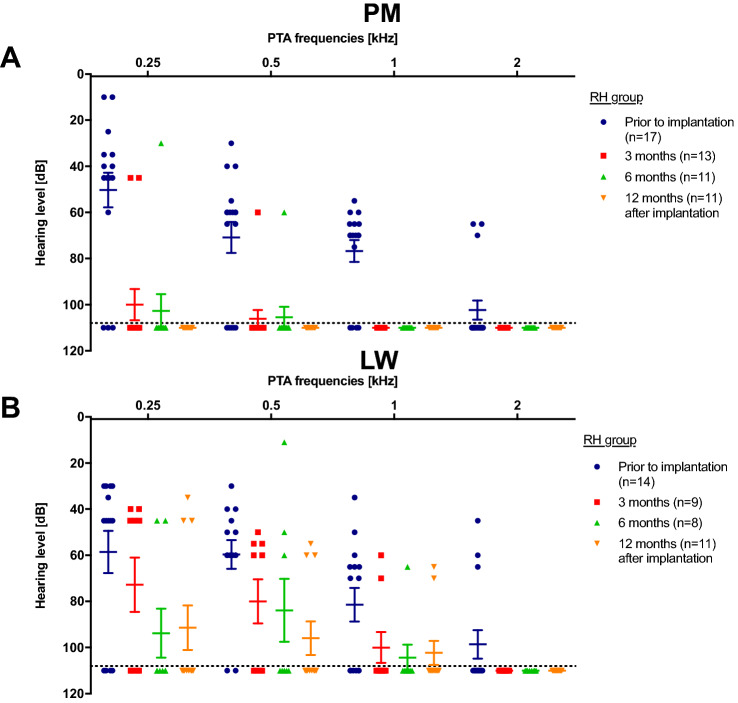
Pure tone audiometry hearing levels **a** PM and **b** LW patients for frequencies between 250 and 2000 Hz. **a** Hearing levels of all PM patients decreased to no residual hearing (> 110 dB) at 12-month follow up. **b** Some LW patients showed preserved residual hearing for lower frequencies (250–1000 Hz) for the first-year follow-up period

Bone-conduction PTA levels in PM-implanted patients rapidly adjusted to noRH values (> 110 dB) in the course of the first 6-month follow-up: PTA averages prior to implantation. After 12 months, none of the implanted patients showed RH anymore (Fig. 2a). However, in the LW group, more patients retained RH compared to the PM group. Specifically, the lower frequencies (250–500 Hz) were more stable (Fig. 2b). None of the patients with HP used Electric Acoustic Stimulation (EAS).

**Fig. 3:**
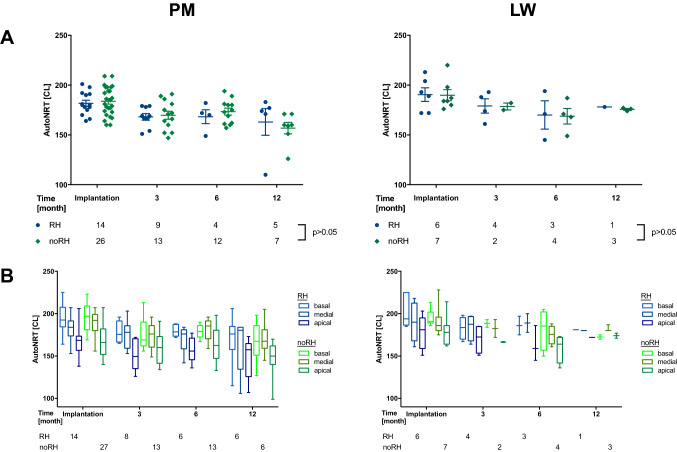
Auto tNRTs for the PM and LW recipients. **a** The average tNRT data showed no significant threshold differences in both RH/noRH groups and equal threshold levels for both PM and LW cohorts. **b** For the sub-locations of the PM and LW electrode, NRT thresholds decreased from basal to apical areas and did not differ between both RH/noRH groups (*p* > 0.05)

Auto threshold (*t*) NRT values showed after an initial decrease at 3-month follow-up stable CL without significant differences between RH and noRH patients in the follow-up period (*p* > 0.05, Fig. 3a). tNRT thresholds were lower for apical than medial or basal localization in the cochlea in both LW and PM recipients (Fig. 3b).

**Fig. 4:**
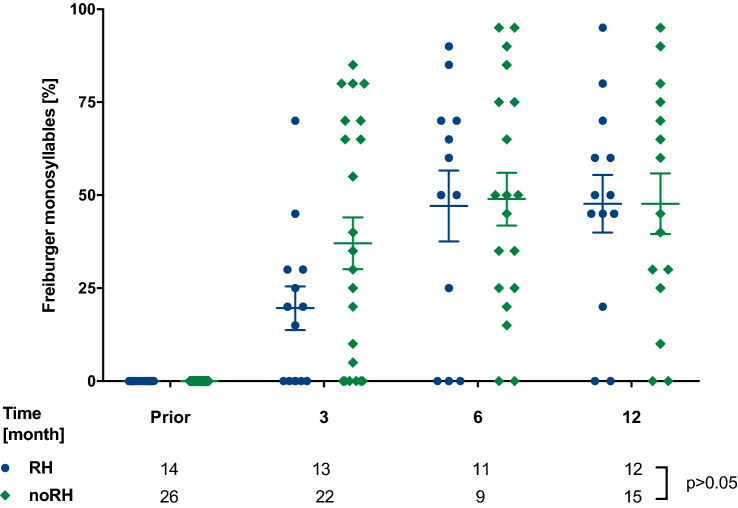
Maximum monosyllabic understanding in Freiburg speech test without any hearing aid prior to surgery and with PM only on the implanted side showed comparable results for both PM/RH and PM/noRH patients (*p* > 0.05)

Maximum Freiburg monosyllabic word understanding at 65 dB measured with PM cochlear implant while masking the contralateral ear showed a similar increase in both PM/RH and PM/noRH, over 12 months (*p* > 0.05, Fig. 4).

**Fig. 5:**
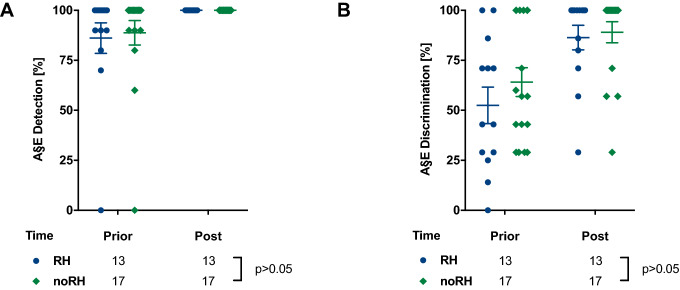
A§E^®^ phoneme **a** detection and **b** discrimination was repeated in best aided condition until 100% was reached during the first-year follow-up period. **a** All PM patients reached 100% detection levels and **b** about 90% discrimination levels with no significant differences between PM/RH and PM/noRH group (*p* > 0.05)

The patients were able to successfully detect and discriminate the A§E^®^ phonemes in best aided condition. Overall, more patients had higher phoneme detection than discrimination levels and the noRH group demonstrated slightly better results than the RH group (*p* > 0.05, Fig. 5).

HISQUI19 indicated an initial subjective increase to a medium/good sound quality with average values of 67/150 to 92/150 points, particularly in the noRH group. Hence, after 12 months, both RH and noRH patients stated a medium sound quality with a mean of 68/150 points (Suppl. Fig. 1a).

Oldenburg Inventory (OI) showed comparable average satisfaction after cochlear implantation in both groups in the first year with slightly higher satisfaction rates in favor of the noRH group. Overall, patients in best aided condition indicated high satisfaction rates of hearing in silence (> 75%) (Suppl. Fig. 1b), whereas hearing in noise and directional hearing reached lower satisfaction rates of 60–75% in both groups (Suppl. Fig. c/d). As an internal control for OI, questions addressing hearing without any hearing aid (wo HA) were included in the questionnaires and patients indicated the same levels (< 25%) as prior to implantation (Suppl. Fig. 1b–d).

## Discussion

This study aims at (i) evaluating HP in PM recipients with RH and (ii) in context of the implanted electrode design of the PM electrode compared to the straight LW electrode. The results indicate that HP is more favorable with the LW electrode than PM electrode. Especially, bone-conducted PTA revealed that all PM recipients lost their RH, whereas approximately 25% LW patients had well-preserved low frequency hearing (250–500 Hz) 1 year after implantation. Overall, HP in PM electrodes has been controversially discussed in the literature. Other investigations demonstrated identical results for both PM and LW electrode groups, and, at 6-month or 1-year follow-up [20, 21]. However, Holder et al. observed better pure-tone averages in PM patients than in LW patients at 6-month follow-up [22]. Also, others observed good HP outcomes among PM recipients [23, 24]. The cochlear location of the PM electrode with a narrower electrode-neuron interface than LW electrodes results in a better HP being expected, however, other factors as surgical technique, experience, cochlear approach and disease-associated progressive hearing loss influence hearing outcomes. Comparative analyses of interaural hearing could help to distinct between disease progression and electrode-induced loss of RH. Snels et al. reported of a symmetric hearing loss in the implanted and contralateral ear in LW recipients. Thus hearing loss was most likely affected by progressive disease [25].

The comparatively better RH levels of LW electrodes are ascribed to the thin and flexible electrode array, which results in less insertion damage. Hence, less neuron damage, fibrosis induction and bone-development is induced [11, 14]. Precurved slim PM electrode arrays tend to have a broader basis requiring for an extended cochleostomy or round window approach causing more insertion trauma particularly in the basal areas of the cochlea. This is mainly due to the mandatory insertion sheath requiring an opening greater than 1 mm, which in turn minimizes the advantage of the slim electrode design (diameter of the tip/apex: 0.4 mm). However, the cochlear approach is less critical for thin LW electrodes [26].

Lately, the monitoring of intracochlear electrocochleographic amplitudes during cochlear implantation has been demonstrated to be beneficial for HP [27, 28].

The Auto tNRT results of the PM group support this hypothesis: Current levels for basal regions are higher than medial or apical regions and do not show significant differences between the RH and noRH group (Fig. 3b). Although the PM electrodes are expected to provide a better neuron-electrode interface, Auto tNRT results showed comparable results compared to the LW electrode (Fig. 3a, b). However, this has been controversially discussed so far: whereas some groups observed NRT levels to be larger with increased distance to the modiolus [29, 30], previous observations made by us and other groups revealed no significant differences [3–6, 31].

Tip fold over was observed in 2 of 47 cases in the PM patients (4.2%) and none in the LW patient group. The tip fold over rate with the PM electrode in this study is roughly two times higher than the tip fold over rate with other electrodes, consistent with a larger case study of 235 CI patients by Friedmann et al. and others [24, 32, 33]. This might be due to the distinct insertion mechanism which is slightly more error-prone on one hand and as always, when using new tools, a certain learning curve is needed even for very experienced surgeons on the other hand. To provide a better final location of PM electrodes the pull-back method has been suggested by Todt et al. [34, 35].

During the first 6 months monosyllabic word recognition in Freiburg speech test showed better results in the noRH group in comparison to the RH group and equalized at 12-month follow-up (Fig. 4). A§E^®^ detection and discrimination showed no significant distinction among both groups (Fig. 5). As expected, phoneme detection is feasible earlier than phoneme discrimination reflecting the results of the Freiburg speech recognition test. These observations of a better phoneme understanding following implantation are supported by Hey et al. and our previous work [19, 36]. HISQUI19 and Oldenburg Inventory showed low participation rates in the follow-up period and might be partly due to loss of follow-up but also reduced willingness or misunderstanding related to the forms. Overall, in HISQUI19 noRH patients seemed to be more satisfied than RH patients during the initial fittings (3–6 months) but equalized after 12 months. This could be caused by lower expectations for the implant by the noRH group (Suppl. Fig. 1a). The Oldenburg Inventory addressed different daily hearing situations: Particularly, in silent conditions patients indicated high satisfaction rates, whereas hearing in noise and directional hearing reached medium satisfaction. This observation is a general hindrance for patients with cochlear implants [37–39]. Both PM RH and noRH patients benefited to a similar extent at the end of the observation period (6–12 months) (Suppl. Fig. 1b–d). The observed increase of the satisfaction rates are congruent with other studies [36] and are up to the expectations from the audiometry results showing no significant difference as well. The limitations of this study were (I) the partly prospective, partly retrospective study design (II) loss of follow-up during observation period, (III) low participation rates in the questionnaires. Still, the results show that electrode design and cochlear access alone seem to have a limited influence on RH, even though LW recipients tended to better HP results than PM recipients at 1-year follow-up. Thus, the electrode design solely cannot be sufficient for HP on long term and other techniques to reduce scar tissue or new technologies, like insertion monitored by electrocochleography, might be necessary in the future.

## Conclusions

The results of the PM recipients revealed that (i) all PM recipients with RH lost their residual hearing within 1 year and (ii) the electrode design had an impact on HP.

## Supplementary Information

Below is the link to the electronic supplementary material.

Supplementary Figure 1. PM patients rated their (A) overall sound quality in HISQUI19 and (B) hearing impression in certain daily situations (silence, noise and directional hearing) in Oldenburg Inventory (OI). (A) noRH patients rated better overall sound quality than RH patients during the initial fittings. Rates equalized at the first-year follow-up (p>0.05). (B) OI-Hearing in silence was high (75-100%) and over 12 months stable ranked in both PM/RH and PM/noRH. (C) Hearing in noise and (D) directional hearing was consistently medium ranked in both groups (p>0.05). Ranking values without any hearing aid reached very low rates as expected and comparable to the rankings prior to implantation

## Data Availability

Data is available upon request.
