# Supplementary figures and images for: Evaluation of hearing preservation in adults with a slim perimodiolar electrode
Source: Eur Arch Otorhinolaryngol. 2021 Apr 8;279(3):1233–42. doi: 10.1007/s00405-021-06755-z (PMC8897335; doi:10.1007/s00405-021-06755-z)

## Slide 1
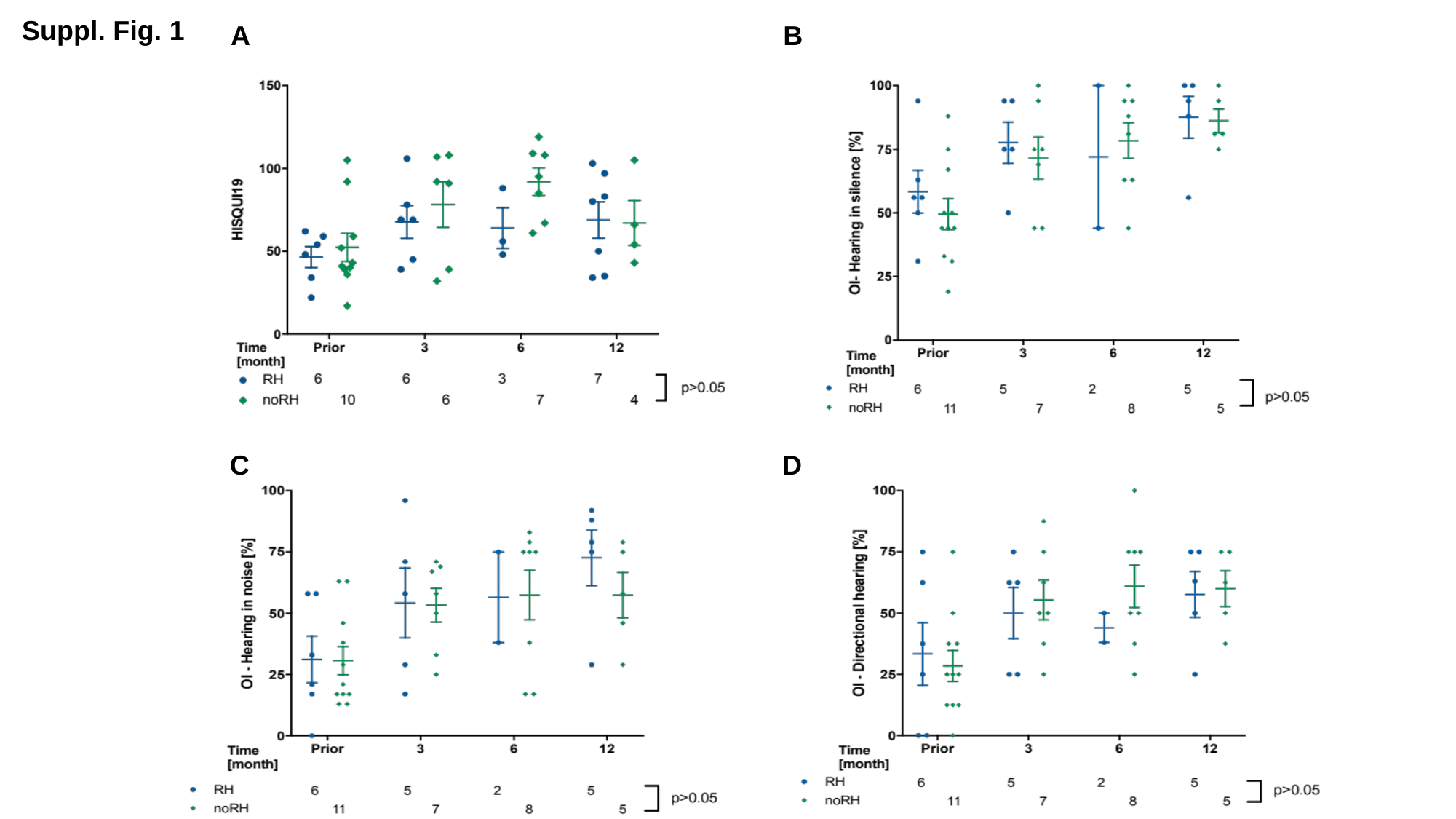

Suppl. Fig. 1
B
A
C
D

Supplement: Supplementary file 1 — Supplementary Figure 1. PM patients rated their (A) overall sound quality in HISQUI19 and (B) hearing impression in certain daily situations (silence, noise and directional hearing) in Oldenburg Inventory (OI). (A) noRH patients rated better overall sound quality than RH patients during the initial fittings. Rates equalized at the first-year follow-up (p>0.05). (B) OI-Hearing in silence was high (75-100%) and over 12 months stable ranked in both PM/RH and PM/noRH. (C) Hearing in noise and (D) directional hearing was consistently medium ranked in both groups (p>0.05). Ranking values without any hearing aid reached very low rates as expected and comparable to the rankings prior to implantation [file 405_2021_6755_MOESM1_ESM.pptx]
